# Protein Arginine Methyltransferase 2 Inhibits Angiotensin II-Induced Proliferation and Inflammation in Vascular Smooth Muscle Cells

**DOI:** 10.1155/2018/1547452

**Published:** 2018-08-13

**Authors:** Si-yu Zeng, Jing-fei Luo, Hai-yan Quan, Yun-bin Xiao, Yu-huan Liu, Hui-qin Lu, Xu-ping Qin

**Affiliations:** ^1^Institution of Drug Clinical Trial, Guangdong Second Provincial General Hospital, Guangzhou, Guangdong 510317, China; ^2^Hunan Province Cooperative Innovation Center for Molecular Target New Drug Study, 28 Western Changsheng Road, Hengyang, Hunan Province 421001, China; ^3^Laboratory of Vascular Biology, Institute of Pharmacy and Pharmacology, University of South China, Hengyang, Hunan 421001, China; ^4^Academy of Pediatrics, University of South China, Changsha, Hunan 410007, China

## Abstract

**Objectives:**

Protein arginine methyltransferase 2 (PRMT2) protects against vascular injury-induced intimal hyperplasia; however, little is known about the role of PRMT2 in angiotensin II (Ang II)-induced VSMCs proliferation and inflammation. This research aims to determine whether PRMT2 inhibits Ang II-induced proliferation and inflammation of vascular smooth muscle cells (VSMCs).

**Materials and Methods:**

PRMT2 overexpression was used to elucidate the role of PRMT2 in Ang II-induced VSMCs proliferation and inflammation. Western blotting and reverse transcriptional PCR were adopted to detect protein and mRNA expression severally. Cell viability was evaluated by 3-(4,5-Dimethylthiazol-2-yl)-2,5-Diphenyltetrazolium Bromide (MTT) assay and cell cycle distribution by flow cytometry.

**Results:**

Ang II significantly reduced mRNA and protein levels of PRMT2 in VSMCs in time-dependent and dose-dependent manner. Results of PRMT2 overexpression indicated that PRMT2 inhibited proliferation of VSMCs stimulated with 100 nmol/L Ang II for 24 hours. Furthermore, overexpression of PRMT2 reduced Ang II-induced production of proinflammatory cytokines such as interleukin 6 (IL-6) and interleukin 1*β* (IL-1*β*) in VSMCs.

**Conclusions:**

These findings suggest that PRMT2 alleviates Ang II-induced VSMCs proliferation and inflammation, providing a new mechanism about how Ang II mediated VSMCs proliferation and inflammation.

## 1. Introduction

Ang II is the primary effector hormone of the renin-angiotensin system that plays a critical role in physiological and pathological processes of cardiovascular system. Not only does it mediate immediate physiological effects such as vasoconstriction and blood pressure regulation, but it also results in cardiovascular diseases (such as hypertension and atherosclerosis) associated with proliferation and inflammation in vascular smooth muscle cells (VSMCs) [1–3]. Even though several studies have been studied for many years about how Ang II induces VSMCs proliferation and inflammation, additional details are still needed to provide potential targets for developing drugs against cardiovascular diseases.

As a key member of protein arginine methyltransferase family, PRMT2 could catalyze transfer of methyl groups from S-adenosylmethionine to arginine residues of substrates proteins. This enzyme contains a highly conserved catalytic Ado-Met binding domain and a unique Src homology 3 domain that binds proteins with proline-rich motifs [4, 5]. Although not initially described to have methyltransferase activity [5], subsequent studies indicate that PRMT2 binds estrogen receptor-*ɑ* and then enhances estrogen-related transcription through its Ado-Met domain, indirectly demonstrating the methyltransferase activity of its own [6, 7]. Depletion of PRMT2 inhibits proliferation of breast cancer cells by suppressing transcriptional activity of cyclin D1 [8, 9]. A series of PRMT2-dependent genes, involved in cell cycle checkpoint and G1/S transition of mitotic cell cycle control, have been identified through WGCNA analysis of PRMT2 signature [9]. Further, vascular injury to PRMT2 (-/-) arteries induced by wire injury leads to intimal hyperplasia [10]. These studies indicate PRMT2 may regulate VSMCs proliferation in cardiovascular system; however, little is known about the role of PRMT2 in Ang II-induced VSMCs proliferation.

*In vivo* and* in vitro* studies show that Ang II induces the expression of proinflammatory cytokines in VSMCs; they have been identified as inflammatory markers including IL-6, IL-1*β*, vascular cell adhesion molecule-1 (VCAM-1), and monocyte chemoattractant protein-1 (MCP-1) [11–13]. PRMT2 loss of function accelerates lipopolysaccharide-induced macrophagic inflammation [14]. No evidence, however, has been provided about the role of PRMT2 in Ang II-induced VSMCs inflammation. In this research, we examined the hypothesis that PRMT2 inhibits cell proliferation and inflammation in VSMCs treated with Ang II.

## 2. Materials and Methods

### 2.1. Cell Culture

Human aortic cell line of VSMCs was originated from ATCC cell bank (Manassas, VA, USA). Cells were cultured with Dulbecco's Modified Eagle's medium (DMEM) containing 10% fetal bovine serum in incubator with 5% carbon dioxide at 37°C. Before stimulated with Ang II or Ang II plus PRMT2 plasmid, cells were needed to incubate for 24 hours in DMEM with 0.1% FBS.

### 2.2. PRMT2 Overexpression

PRMT2 plasmids were constructed in the vector pcDNA3.1 by a commercial service (Sangon, Shanghai, China), while pcDNA3.1 vectors were adopted as the control. PRMT2 plasmids or control plasmids (8 *μ*g) were transiently transfected into VSMCs by adding themselves to 15 *μ*L lipofectamine 2000 (11668019, Thermo Scientific, Shanghai, China) per 21 cm^2^ dish. These cells were stimulated with lipofectamine 2000, or Ang II, pcDNA3.1 vectors, and lipofectamine 2000, or Ang II, PRMT2 plasmids, and lipofectamine 2000.

### 2.3. Total Protein Extraction

Cell protein was extracted using RIPA lysis buffer (P0013B, Beyotime Institute of Biotechnology, Jiangsu, China), including 50 mM Tris (pH 7.4), 150 mM sodium chloride, 1% (wt/vol) Triton X-100, 1% (wt/vol) sodium deoxycholate, and 0.1% (wt/vol) sodium dodecyl sulfate. Phenylmethane sulfonyl fluoride (PMSF; ST506, Beyotime Institute of Biotechnology, Jiangsu, China) should be added to the lysis buffer before application with a final concentration of 1 mmol/L. After cells were incubated with RIPA lysis buffer for 30 minutes on ice, the solution was centrifuged at 14000 g for 5 minutes to remove cell debris. And then the protein concentration of the supernatant was determined using BCA protein assay kit (P0010, Beyotime Institute of Biotechnology, Jiangsu, China). Finally, equal total content of protein was used for western blotting.

### 2.4. Western Blotting

Cell protein was separated in SDS gel electrophoresis, transferred to polyvinylidine difluoride membranes (PVDFs), and then incubated with primary antibody overnight at 4°C. These following primary antibodies were used, respectively: PRMT2 antibody (ab66763, Abcam, MA, USA), proliferating cell nuclear antigen (PCNA) antibody (ab152112, Abcam, MA, USA), interleukin 6 antibody (12153, CST, MA, USA), interleukin 1*β* antibody (12703, CST, MA, USA), and *β*-actin (SC4778, Santa Cruz, TX, USA). These PVDFs were then incubated with secondary antibody conjugated with horseradish peroxidase. Relative levels of immunoreactive protein were detected by chemiluminescence and then quantified with Image J software.

### 2.5. Reverse Transcription PCR

Reverse transcription PCR was carried out as described previously [15]. Primers for PRMT2 and GADPH were designed and synthesized by Sangon (Shanghai, China). Primer for PRMT2: 5′-AGAAGGCTGGGGCTCATTTG-3′ (forward primer), 5′-AGGGGCCATCCACAGTCTTC-3′ (reverse primer); Primer for GADPH, 5′-ACCACAGTCCATGCCATCAC-3′ (forward primer), 5′-TCCACCACCC TGTTGCTGTA-3′ (reverse primer). Total cellular RNA was extracted using Trizol, and then it was used to synthesize the first-strand complementary DNA using reverse transcription kit (4387406, Thermo Scientific, Shanghai, China). PCR amplification profiles were described as follows: 94°C for 2 minutes, 35 cycles of 94°C for 30 seconds, 55°C for 30 seconds and 72°C for 30 seconds, and finally 72°C for 2 minutes. Equal amount of both PRMT2 and GADPH RT-PCR products was loaded and then separated on 2% agarose gels. Optical densities of ethidium bromide-stained DNA bands were quantitated, and the results were expressed as ratio of PRMT2 to GADPH (PRMT2 /GADPH).

### 2.6. Assay of 3-(4,5-Dimethylthiazol-2-yl)-2,5-Diphenyltetrazolium Bromide (MTT)

MTT assay was utilized to measure cell viability as described previously [15]. It is briefly described as follows: first, VSMCs were continuously incubated for 4 hours in cell media with 10 *μ*L MTT (5g /L); second, cells were incubated with 150 *μ*L DMSO for 10 minutes at room temperature; third, the optical density (OD) of VSMCs was measured under 490 nm by automatic enzyme-linked immune-adsorbent assay system.

### 2.7. Cell Cycle Distribution

Flow cytometry was adopted to evaluate and analyze cycle distribution of VSMCs as described previously [15]. Firstly, cells were harvested via trypsin digestion, washed with precooled phosphate buffered saline (PBS), and then fixed for 24 hours using precooled 75% ethyl alcohol. Next, these cells were coincubated with 100 *μ*L RNase A for 30 min at 37°C and then with 500 *μ*L staining liquids for 30 min at 4°C when avoiding light. Finally, these cells were assayed by flow cytometer to determine cells percentage in each phase of cell cycle. At least 5000 cells were counted for each analysis.

### 2.8. Statistical Analysis

Data were expressed as means ± SD. Statistical significance was determined by unpaired Student's t-test between two groups and by one-way or two-way ANOVA among at least three groups followed by Bonferroni's post hoc test (Prism 7.0; GraphPad Software, CA, USA), and the value of* P*<0.05 (two-sided) was considered significant.

## 3. Results

### 3.1. Ang II Decreased PRMT2 Expression in Proliferative VSMCs

**Figure 1:**
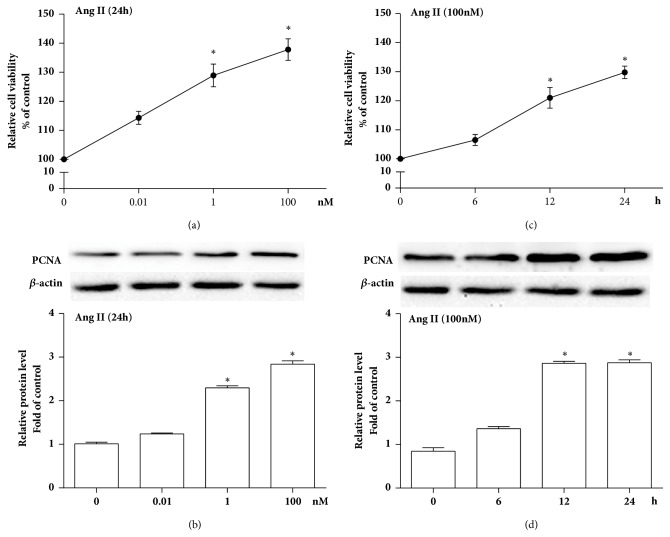
Ang II induced VSMCs proliferation in a time-dependent and dose-dependent manner. (a-b) Dosage curve of cell proliferation when cells were treated with different concentrations of Ang II for 24 hours. (a) Cell viability; (b) PCNA protein level; ^*∗*^*P*<0.05 versus 0 nM. (c-d) Time curve of VSMCs proliferation when cells were treated with 100 nM Ang II. (c) Cell viability; (d) PCNA protein level; ^*∗*^*P*<0.05 versus 0 h. Ang II represents angiotensin II; VSMCs represent vascular smooth muscle cells; n=3 independent experiments.

**Figure 2:**
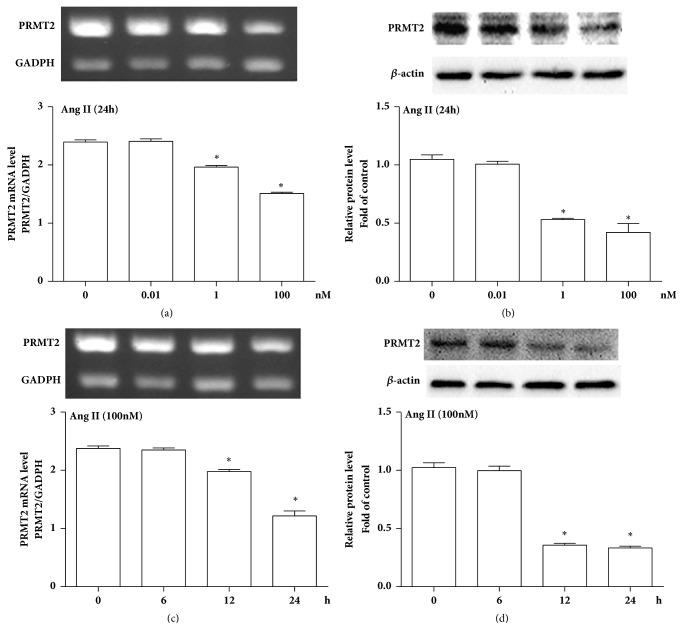
Ang II reduced PRMT2 expression in a time-dependent and dose-dependent manner. (a-b) Dosage curve of PRMT2 expression when cells were stimulated with different concentrations of Ang II for 24 hours. (a) PRMT2 mRNA level; (b) PRMT2 protein level; ^*∗*^*P*<0.05 versus 0 nM. (c-d) Time curve of PRMT2 expression when cells were stimulated with 100 nM Ang II. (c) PRMT2 mRNA level; (d) PRMT2 protein level; ^*∗*^*P*<0.05 versus 0 h. Ang II represents angiotensin II; VSMCs represent vascular smooth muscle cells; n=3 independent experiments.

As shown in Figure 1, Ang II promoted VSMCs proliferation, indicated by the increase of cell viability and PCNA protein level, in VSMCs stimulated with 100 nmol/L Ang II for 12 and 24 hours, or different concentration of Ang II such as 1 and 100 nmol/L. Concurrently, Ang II significantly reduced mRNA and protein levels of PRMT2 in VSMCs in time-dependent and dose-dependent manner (Figure 2). These parallel results suggested that Ang II diminished PRMT2 expression in proliferative VSMCs.

### 3.2. PRMT2 Protected against Ang II-Induced VSMCs Proliferation

**Figure 3:**
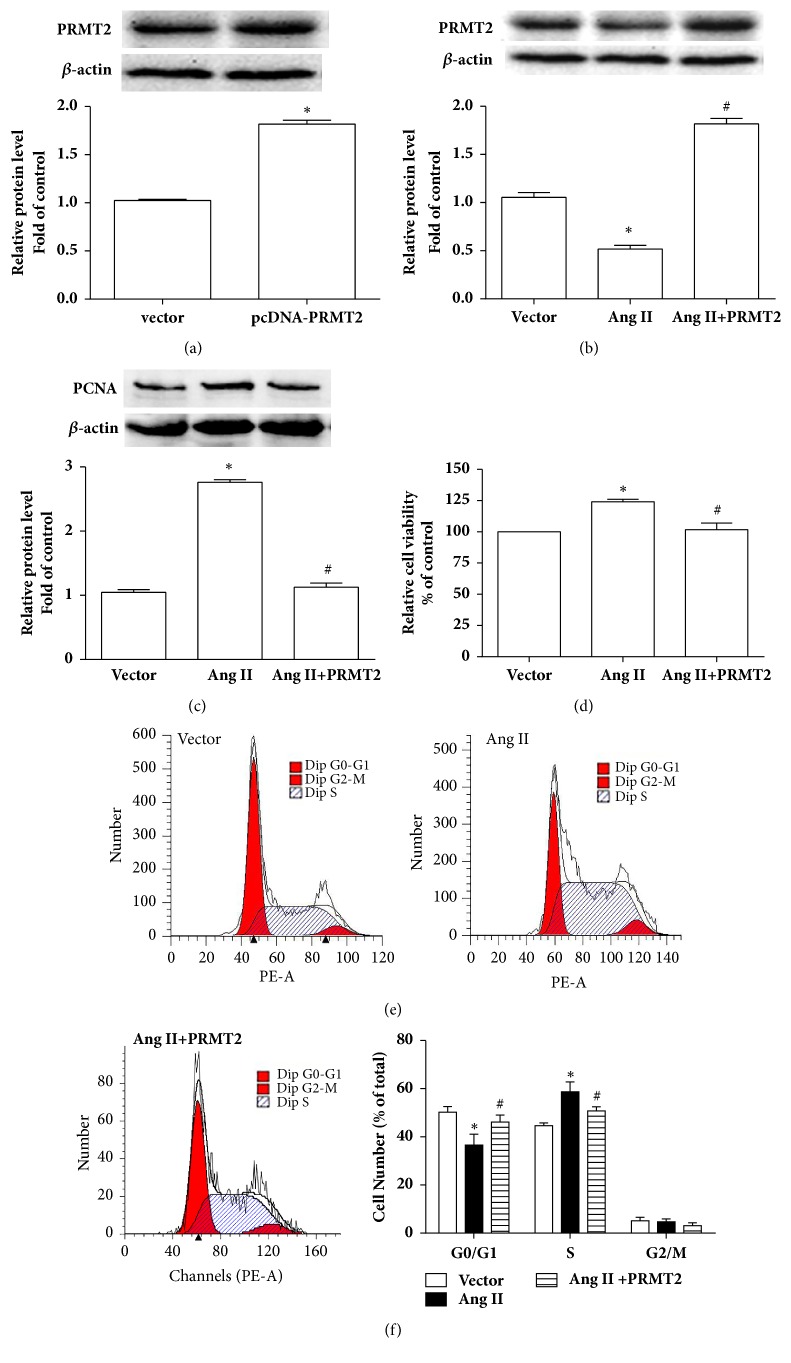
PRMT2 overexpression attenuated Ang II-induced VSMCs proliferation. (a) PRMT2 protein level. Cells were transfected with pcDNA3.1-PRMT2 for 48 hours. (b-f) After transfected with pcDNA3.1-PRMT2 for 24 hours, VSMCs were treated with 100 nM Ang II for 24 hours. (b) PRMT2 protein level; (c) PCNA protein level; (d) cell viability; (e) representative results of cell cycle distribution; (f) cell number. Ang II represents angiotensin II; VSMCs represent vascular smooth muscle cells. ^*∗*^*P*<0.05 versus vector group, ^#^*P*<0.05 versus Ang II group. n=3 independent experiments.

PRMT2 overexpression was used to determine the effect of PRMT2 on Ang II-induced VSMCs proliferation. As shown in Figure 3(a), PRMT2 protein level was significantly elevated in VSMCs transfected with pcDNA3.1-PRMT2 for 48 hours compared with vector group, demonstrating the reliability of PRMT2 plasmid. In accordance with results of Figure 1, VSMCs were treated with 100 nmol/L Ang II for 24 hours to induce VSMCs proliferation. Figures 3(b), 3(c), and 3(d) showed that PRMT2 overexpression remarkably decreased PCNA protein level and cell viability induced by Ang II. Results of flow cytometry indicated that Ang II stimulation reduced the proportion of G_0_/G_1_ phase cells but increased that of S phase cells compared with vector group, whereas PRMT2 overexpression reversed Ang II-induced changes in proportion of G_0_/G_1_ and S phase cells (Figures 3(e) and 3(f)). Thus, PRMT2 overexpression could attenuate Ang II-induced VSMCs proliferation.

### 3.3. PRMT2 Inhibited Ang II-Induced VSMCs Inflammation

**Figure 4:**
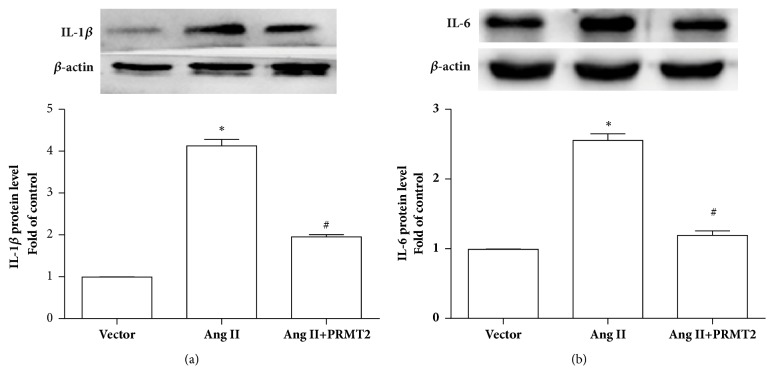
PRMT2 overexpression suppressed Ang II-induced VSMCs inflammation. After transfection with pcDNA3.1-PRMT2 for 24 hours, VSMCs were treated with 100 nM Ang II for 24 hours. (a) IL-1*β* protein level. (b) IL-6 protein level. Ang II represents angiotensin II; VSMCs represent vascular smooth muscle cells. ^*∗*^*P*<0.05 versus vector group, ^#^*P*<0.05 versus Ang II group. n=3 independent experiments.

In addition to leukocytes, VSMCs can be another crucial source of proinflammatory cytokines in the vessel wall [11, 12]. Proinflammatory cytokines such as tumor necrosis factor *α* (TNF*α*) and IL -6 have been recognized as markers of inflammation [16]. As shown in Figure 4, protein levels of IL-1*β* and IL-6 were markedly elevated in Ang II group compared with control group, whereas upregulation of IL-1*β* and IL-6 protein levels was reversed by PRMT2 overexpression. Therefore, PRMT2 prevented Ang II-induced VSMCs inflammation.

## 4. Discussion

In this research, we reported two interrelated discoveries: (1) PRMT2 protected against Ang II- induced VSMCs proliferation, and (2) PRMT2 inhibited Ang II-induced inflammation.

Ang II is a critical mediator that induces VSMCs proliferation through AT1 receptor activation [17]. Activation of mitogen-activated protein kinase (MAPK), including extracellular signal-regulated protein kinase 1/2 (ERK1/2), c-Jun N-terminal kinase (JNK), and p38 MAPK, is required in Ang II-induced VSMCs proliferation [18]. Krüppel-like factor (KLF5), a downstream signal of ERK1/2 and p38 MAPK, activates Ang II-induced VSMC proliferation through cyclin D1 gene transcription via functional interaction with c-Jun [19]. Phosphatidylinositol-3 kinase (PI3K) activation mediates Ang II-induced VSMCs proliferation via ERK1/2 activation [20]. These findings show that MAPK signaling pathway plays a key role in Ang II-induced VSMCs proliferation.

Besides MAPK signaling pathway, oxidative stress, activation of nuclear transcriptional factors, and other signaling pathways also mediated Ang II-induced VSMCs proliferation. Transforming growth factor-*β* (TGF-*β*) signaling by TGF-*β* receptor (through Smad2/3 pathway), Src-dependent epidermal growth factor receptor (EGFR) activation, and NADPH oxidase-induced elevation of reactive oxygen species formation promote Ang II-induced VSMCs proliferation [1, 21, 22]. Nuclear transcriptional factors cAMP response element-binding protein (CREB) and nuclear factor kappaB (NF-kB) could induce Ang II-induced VSMCs proliferation [23, 24]. Further, complement 3a (C3a), Fat1 (an atypical cadherin), and notch signaling pathway contribute to Ang II-induced VSMCs proliferation [22, 25, 26].

In recent years, researchers are paying more and more attention to epigenetic mechanisms that cause Ang II-induced VSMCs proliferation. Recent epigenetic research mainly focuses on noncoding RNA (such as microRNA and long noncoding RNA) and DNA methylation. It has been reported that microRNA-130a, microRNA-761, and microRNA-155 could promote Ang II-induced VSMCs proliferation [27–29]. Protein arginine methyltransferase PRMT2 participates in posttranslational modification through arginine methylation of histones, RNA binding proteins, and transcriptional factors [5, 7, 30]. Our results showed PRMT2 overexpression protected against Ang II-induced VSMCs proliferation, thereby helping better understand the signaling network of Ang II-induced VSMCs proliferation.

NF-*κ*B activation and toll-like receptor 4 (TLR4) activation play crucial role in Ang II-induced VSMCs inflammation. NF-kB and CREB promotes Ang II mediated production of TNF-*α* and IL-6 in VSMCs treated with Ang II [24, 31, 32]. NF-*κ*B, CREB, and ERK-dependent histone acetylation mediated by p300 and steroid receptor coactivator-1 (SRC-1) are also required in Ang II-induced upregulation of IL-6 expression [33]. Results of TLR4 inhibitor, antibody, and siRNA indicate that TLR4 activation plays a key role in Ang II-induced VSMCs inflammation [34, 35]. Our present findings showed that PRMT2 negatively mediated Ang II-induced VSMCs inflammation, supplementing a new mechanism of VSMCs inflammation induced by Ang II.

## 5. Conclusion

In summary, the present study reveals that PRMT2 could inhibit Ang II-induced proliferation and inflammation of VSMCs. This will help us better understand underlying mechanisms of Ang II-induced VSMCs proliferation and inflammation, providing further basis for PRMT2 as a potential target against cardiovascular diseases associated with VSMCs proliferation and inflammation.* In vivo* research, however, is still needed to examine whether PRMT2 mediates VSMCs proliferation and inflammation and vascular remodeling in Ang II-induced hypertensive model.

## Data Availability

The data used to support the findings of this study are available from the corresponding author upon request.
